# The Gray Nine and Parallel Personality Patterns: Big Five, Dark Tetrad, and a “Well-Rounded Personality”

**DOI:** 10.1007/s12124-024-09842-y

**Published:** 2024-05-04

**Authors:** Björn Boman

**Affiliations:** Stockholm University, 114 19, Stockholm, Sweden

**Keywords:** Gray nine, Big five, Dark tetrad, Dark triad, Well-rounded personality

## Abstract

The vast literature on personality psychology generally focuses on neutral or socially beneficial personality traits such as the Five-Factor model (e.g., agreeableness, conscientiousness) or “dark” traits such as Machiavellianism, narcissism, psychopathy, and everyday sadism. However, the current synthesis of the literature indicates that the distinction between benign, malign, and neutral personality traits and facets is partly misguided. In fact, there are many objective and subjective measures that indicate that high agreeableness is not beneficial, while moderate grandiose narcissism is. Many, if not all of the traits are rather gray and socially and personally desired outcomes indicate that people who aim for a well-rounded personality should typically be clustered in the middle of the various personality spectrums. In addition, many of the personality traits are characterized by parallel patterns of good/bad relations to social and personal outcomes.

## Introduction

In personality research there are two personality models that, in tandem, cover a substantial and comprehensive part of human personality dimensions: The Five-Factor Model (i.e., Big Five, openness to experience, conscientiousness, extraversion, agreeableness, neuroticism) and the Dark Tetrad (Machiavellianism, narcissism, psychopathy, everyday sadism). Whereas the Big Five model covers neutral or even benign aspects of human personality and behavior (John & Srivastava, 2009), the Dark Tetrad includes malign traits that are usually associated with anti-social behaviors and proclivities (Buckels et al., 2013; Muris et al., 2017; Truhan et al., 2020).

However, much research indicates that many of these traits, even the supposed dark ones, are neutral and “gray” rather than unitarily dark and malign. While the separation of the Big Five from the Dark Triad and benign and pro-social Light Triad (e.g., Lukic, & Zivanovic, 2021) is still relevant, I argue here for the separation of narcissism from the Dark Tetrad, as well as for the separation of grandiose/overt narcissism from vulnerable/covert narcissism. I will also argue that many of these traits typically require medium rather than high and low scores in order to cultivate an “optimal” and well-rounded personality profile. As such I continue the argument, which was accentuated by Carter et al. (2018) but go a few steps further and expand the theoretical examination to also include the Dark Tetrad. Moreover, the investigation includes instances from political science and political philosophy, thus transcending Jonason et al. (2012), who used examples from popular culture regarding the Dark Triad.

Moreover, to have a so-called well-rounded personality or character is described in official national curricular documents in many educational systems around the world (e.g., Boman & Mosesson, 2023). Creativity, competence, knowledge, and interpersonal “soft” skills, often in conjunction with a humanitarian and global citizenship mindset, are underscored as desired elements within such a personal development (e.g., Boman & Mosesson, 2023; Heckman & Kautz, 2014). For instance, the English version of Sweden’s national curriculum Lgr 11 (Swedish National Agency for Education, 2018, p. 8) stresses that “The school should stimulate pupils’ creativity, curiosity, and self-confidence, as well as their desire to translate ideas into action and solve problems. Pupils should have the opportunity to take initiatives and assume responsibility, and to develop their ability to work both independently and together with others.” Taken at face value, it seems that elements which are related to the Big Five personality traits openness to experience (“curiosity”, “creativity”), extraversion (“self-confidence”, “work both independently and together with others”), and conscientiousness (“responsibility”, “translate ideas into actions and solve problems”) are implicitly, but perhaps undeliberate, expressed in these sentences. A similar personality parlance has been identified in other Western countries, as well as East Asian school systems such as South Korea (Boman & Mosesson, 2023). According to Merriam-Webster (2024), the adjective well-rounded refer to for example “schools that turn out well-rounded graduates”. According to Cambridge Dictionary (2024), well-rounded can be exemplified by “She describes herself as a “well-rounded individual” who works hard but has a varied social life”. In the latter instance, the balance between different behaviors and personality styles is underscored. However, the links between the notions of a well-rounded personality profile and personality research is lacking.

The aim of the current investigation is, therefore, to create a theoretical synthesis of the existing literature which might, to some degree, aid students and people in general to prepare themselves for the real world, instead of a politically correct version of an ideal person existing in an imaginary realm. Such a synthesis accentuates many of the gray areas and components of personality and behavior in relation to real-world social outcomes, both objective (academic achievement, job performance, income) and subjective (stress, well-being). The analysis does also consider Aristotle’s concept of *sophrosyne*, the responsible mean, and *eudaimonia* as analytical tools (e.g., Aristotle, 1999a, b; Macintyre, 1998; Seligman, 2002). However, these analytical tools are only relevant in so far as they aid in the understanding or application of personality traits and the various, often medium levels of these that are often required to live a fulfilled life (i.e., *eudaimonia*). Furthermore, some examples from popular culture and politics will also be provided in some sections.

It is important to underscore that the “ideal type” individual which is described in later sections is nothing more than a typological description of how to think about the extent to which different personality traits are required and can be balanced in relation to various social and personal outcomes in life, as well as other individuals and social structures. One of the key findings in personality psychology and developmental psychology is that individuals differ substantially (John & Srivastava, 2009). No one expects individuals to be or become identical whatsoever. The author does not argue for normative sameness. Neither are situations and contexts ignored. In certain situations and contexts are, for example, a very high level of agreeableness and extraversion particularly beneficial for oneself (e.g., job types). Moreover, at the group level different personality profiles may complement each other (e.g., Lykourentzhou et al., 2016). Nevertheless, typologies can be useful to highlight important psychological and social aspects and patterns. In this regard, the main focus is on individual typologies rather than group dynamics typologies.

In addition, the theoretical synthesis transcends the examination of gradual personality differences in relation to socially and personally beneficial associations as it also focuses on the parallel patterns of good/bad in relation to these nine personality traits (Boman, 2024, p. 82).

## Theoretical Background

### The Core of the Big Five and Dark Tetrad

The Five-Factor Model or the Big Five has its roots in linguistic psychology (Goldberg, 1990) but, to an extent, also in psychoanalysis. For example, Sigmund Freud’s idea of the super-ego (*das Über-ich*) has influenced conscientiousness (Roberts et al., 2013). Freud also wrote extensively on neurosis (e.g., Freud, 1990; Rosenfeld, 1990). The Big Five consists of traits – openness to experience, conscientiousness, extraversion, agreeableness, and neuroticism – and these consist of, usually, six sub-traits (i.e., facets). Moreover, some of the traits might also be described as mirroring an opposite trait: negative scores on extraversion items imply introversion, and low scores on neuroticism items imply emotional stability.

Starting at the trait level, people who are high in openness are imaginative, creative, and curious, whereas conscientious persons are being organized, goal-oriented, and self-disciplined. Extroverts are active, assertive, and desiring social relationships. Agreeable individuals are tenderminded, altruistic, and trustworthy, while neurotic individuals are anxious and emotionally unstable (e.g., Alderotti et al., 2023). At the facet level, there is no complete consensus regarding which facets that appropriately represent the Big Five factors. However, many constructs include fantasy, esthetics, feelings, action, ideas and values for openness, competence, order, dutifulness, achievement striving, self-discipline and deliberation for conscientiousness, warmth, gregariousness, assertiveness, activity, excitement seeking, and positive emotions for extraversion, trust, straightforwardness, altruism, compliance, modesty, and tendermindedness for agreeableness, and anxiety, angry hostility, depression, self-consciousness, impulsiveness, and vulnerability for neuroticism (Ziegeler et al., 2014). The Big Five are usually measured with a 44-item Likert scale test (e.g., Costa & McCrae, 1992) but also with longer and shorter versions. For example, the brief Big-Five Inventory-10 (BFI-10) consists of items such as “I see myself as someone who is reserved”, “…is generally trusting”, and “…does a thorough job”. Two items represent each one of the five traits and therefore the shorter scales do not cover the facets particularly well (Rammstedt & John, 2007).

The core of the Dark Tetrad are the sub-clinical traits Machiavellianism, narcissism, psychopathy, and everyday sadism. These share some features such as relative callousness but only psychopaths are associated with criminality whereas only sadists enjoy cruelty (Paulhus, 2014). Machiavellians are associated with manipulation, immorality, and a cynical worldview. Narcissism is associated with a sense of entitlement, exhibitionism (e.g., discursive, physical) and a very high valuation of one’s skills and achievements. Narcissism does both have an agentic side and a more defensive side related to ego threatening aspects. Psychopathy is linked with aggressiveness, impulsivity, irresponsibility, and socially aversive behaviors, while sadists are characterized as enjoying the harm of others and an aggressive behavioral repertoire. The SD4 consists of seven items for each trait of the Dark Tetrad (Böckels et al., 2021).

### Personality Traits and Adaptation

An important viewpoint is the evolutionary adaptiveness of traits that are considered benign, neutral, or intuitively maladaptive (e.g., Carter et al., 2018; Jonason et al., 2009; MacDonald, 1998). This perspective does also support the evolutionary “diffusion” of various kinds of personality traits, whether they are socially acceptable or not. Personality traits are partly heritable, which is evident by twin and adoption studies and many agree that the evolution of personality traits is not neutral. However, the links between number of offspring and personality traits are relatively weak and inconclusive (Penke & Jokela, 2016).

Thus, a second dimension to consider, and as such agnostic about reproductive success (i.e., intergenerational adaptation), is how personally and socially “adaptive” a trait is in a particular society. In other words: How can a certain personality trait or profile aid or prevent a person in his or her life? (Carter et al., 2018). Regardless of the actual number of offspring among those who are high or low in for example neuroticism, extraversion, or narcissism, these traits could be more less beneficial in one’s personal and social life.

In that regard, it is easy to agree with Carter et al. (2018) that more is not always better in terms of the Big Five (i.e., the notion that a high degree of these traits is beneficial). Instead, a moderate degree of openness, conscientiousness, extraversion, agreeableness, and emotional stability seems to be generally beneficial. However, as will be highlighted in the next and later sections this moderate degree–personally/socially beneficial relation seems to be partly extended also to the Dark Tetrad. At least, considerable nuance is required regarding these so-called dark traits. The Big Five is essentially a descriptive personality theory and its proponents do not imply that personality traits are good or bad. However, it might be the case that many regard at least (high) agreeableness, conscientiousness, and emotional stability as socially beneficial traits due to their inherent characteristics and external validity (Carter et al., 2018).

### Research on the Big Five, Dark Triad/Tetrad and Social Outcomes

Research on the Big Five, especially at the trait level, is abundant. Studies include relations between the Big Five and academic achievement (e.g., Poropat, 2009; Mammadov, 2022), associations between the Big Five and Dark triad (e.g., Paulhus & Williams, 2002) and Dark tetrad (Buckels et al., 2013), cross-cultural gender differences on the Big Five (e.g., Kajonius & Johnson, 2018; Schmitt et al., 2009; Weisberg et al., 2011), as well as relations between Big Five and intelligence (Duckworth et al., 2012), and personality and job performance (e.g., Salgado, 1998; Witt et al., 2002). Longitudinal studies that focus on how personality changes over the life span are also comprehensive (e.g., Caspi et al., 2005; Roberts et al., 2008).

Many studies show that most of the Big Five traits have only small or moderate links to both negative and positive social outcomes. For example, agreeableness, extraversion, and openness to experience are rather weak predictors of academic achievement (Mammadov, 2022) and job performance (Salgado, 1998). However, both conscientiousness and emotional stability (i.e., the opposite of neuroticism) are associated with academic achievement (Andersen et al., 2020; Mammadov, 2022, Poropat, 2009) and job performance (Barrick & Mount, 2012; Salgado, 1998; Snyder & Lopez, 2002). In fact, the combination of high cognitive ability, conscientiousness, intellectual curiosity, and emotional stability is a strong recipe for academic and occupational success (Barrick & Mount, 2012; Mammadov, 2022; von Stumm et al., 2011). While there are some studies that emphasize the real or potential downsides of having a very high level of conscientiousness (e.g., Coleman et al., 2022), conscientiousness alongside emotional stability can be considered predominantly positive (i.e., good for the individual) and pro-social (i.e., good for society) traits. If the prescriptive partly follows the descriptive, an individual should thus aim to be or become more conscientious and emotionally stable.

Agreeableness is a pro-social personality trait which is negatively associated with the Dark Triad/Tetrad (Buckels et al., 2013; Paulhus & Williams, 2002). It has typically small positive correlations with academic achievement (Mammadov, 2022), job performance (Witt et al., 2002; Ziegeler et al., 2014), but generally not income in adult populations (Duckworth et al., 2012; Matz & Gladstone, 2020). However, these effect sizes for agreeableness are smaller than conscientiousness in relation to both job performance and academic achievement. Moreover, regarding academic achievement the effect sizes for agreeableness and openness to experience decrease virtually to zero when cognitive ability and conscientiousness are included in the same regression models (e.g., Boman, 2023a; Mammadov, 2022). Openness to experience and extraversion resemble agreeableness in that they might have some positive objective socioeconomic outcomes (e.g., Alderotti et al., 2023) but the relations are not as accentuated and sometimes show negative correlations in various domains (Duckworth et al., 2012). Extraversion is positively associated with good outcomes in sales and management professions (Blickle et al., 2015) but less so in other occupational orbits (Ziegeler et al., 2014).

Research on psychopathology has been ongoing for many decades. Narcissism and psychopathy in particular have long roots in, for example, the writings of Sigmund Freud (e.g., Freud, 2014). Specifically, however, Paulhus, Delroy and Wiliams (2002) invented the Dark Triad scale to create a sub-clinical construct that includes three moderately inter-correlated traits: Machiavellianism, narcissism, and psychopathy. Influences were in that regard derived from Christie and Geis’s (1970) work on Machiavellianism, Raskin and Hall’s (1979) work on narcissism, and Hare’s (1985, 1991) work on psychopathy. Several years later, Buckels et al. (2013) showed that (everyday) sadism conceptually and empirically seem to belong to the dark cluster, which implies that the Dark Triad was thereafter extended to a Dark Tetrad (Paulhus, 2014). Nevertheless, research only on the Dark Triad scales is still ongoing, in part because of its measurement brevity (e.g., Jonason & Webster, 2010). On the other hand, there is also a short version for the Dark Tetrad, SD4 (e.g., Blötner et al., 2021).

There are many studies that focus on isolated traits and as such not as being part of the Dark Triad or Dark Tetrad constellations. Beginning with narcissism, many studies show that there are two types of narcissism: an overt and grandiose form and a covert and vulnerable counterpart. These sub-types of narcissism have small inter-correlations and represent two discrete constructs in non-clinical research contexts. However, at the intra-individual level the same person can fluctuate between grandiosity and vulnerability. Hence, the social and personality psychology literature does not necessarily correspond particularly well to clinical literature in that respect (Krizan & Herlache, 2018; Pincus & Roche, 2011). Miller and Maples (2011) highlight moderate positive correlations between grandiose narcissism and the conscientiousness facets competence (0.23) and achievement striving (0.27), as well as negative correlations with neuroticism facets such as vulnerability (-0.30), depression (-0.26) and anxiety (-0.25). Persons with relatively higher degrees of grandiose narcissism are also more extrovert on average. An almost mirror opposite of these tendencies is the case of vulnerable narcissism which is closely related to neuroticism (Miller & Maples, 2011). This line of research has later been corroborated by Furnham and Crump (2014) and Papageorgiou et al. (2019), whose study show that grandiose narcissism is associated with higher subjective well-being and an ability to cope with depression. People with average to high levels of grandiose narcissism might be described as thick-skinned and prone to being emotionally stable, except for the neuroticism facet angry hostility, typically when their egos are being threatened (Miller & Maples, 2011). No such positive social outcomes, whether objective or subjective, have been found for vulnerable narcissism (Miller & Maples, 2011; Papageorgiou et al., 2019). Moreover, narcissism is generally considered to be relatively normal in current times, at least in the West (Rauthmann & Kolar, 2012).

In addition, there are objective associations between narcissism and social outcomes. For example, using large samples from China, Greece and UK, Lu et al., (2023) replicated earlier studies such as Jonason et al. (2018), Paleczek et al. (2018) and Spurk et al. (2016) in that they demonstrated that people who score relatively higher in subclinical grandiose narcissism have higher earnings. The effect sizes are typically small to moderate in this regard. The same relations for Machiavellianism and psychopathy are inconclusive and context-dependent (Luo et al., 2023).

To a degree, sub-clinical psychopathy manifests similar correlation patterns as sub-clinical narcissism (Paulhus & Williams, 2002) but according to later research it is also more closely related to everyday sadism (Bonfa-Araujo et al., 2022; Book et al., 2016). A meta-analytical review by Bonfa-Araujo et al. (2022) indicate that everyday sadism is related to malign and anti-social behaviors and proclivities such as aggressiveness, cyber bullying, lack of empathy, and exploitative mating strategies.

As for Machiavellianism and psychopathy, these are considered more socially and personally undesirable than narcissism (Rauthmann & Kolar, 2012) and generally associated with anti-social behavior. Moreover, these two traits are closely linked to everyday sadism (Bonfa-Araujo et al., 2022).

### Personality, Popular Culture, and Politics

The negative descriptions and connotations of the Dark Tetrad beg the question whether there is anything good at all about these personality traits beyond personal gains under some circumstances. Anti-social traits and behaviors indicate that they are linked with negative social outcomes. In this respect, however, it is important to consider two aspects. From popular culture, one may consider several fictional characters with Dark Triad and Dark Tetrad tendencies (Boman, 2020; Jonason et al., 2012). For example, the fictional special agent James Bond seems to possess both grandiose narcissistic self-love, Machiavellian cunning, and psychopathic callousness. He is a killer by profession and is entitled to not have to abide by the law. That might, at first glance, appear to be the ultimate Dark Tetrad person. Yet Bond is a good guy, and it is rather the specific circumstances and his particular profession as a special agent that enable him to transcend the regular laws. Macintyre (1998, pp. 68–72) underlines that one of the problems with Aristotle’s virtue ethics is that it does not differentiate between contexts and specify which acts that may deem an individual unworthy of praise. But, indeed, Bond and other action heroes are courageous and manifest many other such qualities (e.g., intelligence, wisdom, wittiness). They are also very popular among the mainstream audiences, despite the dark traits which they seem to possess (Jonason et al., 2012).

Other pertinent examples from the popular culture realm is *50 Shades of Grey* (2011), a novel written by E.L. James, which also has two sequels and was made into a film that was released in 2015. The novels in particular became extremely popular throughout the West and beyond, especially among female readers. Christian Grey (the name and the title of the trilogy is probably no coincidence), while complex and dynamic as a character, possesses sadistic tendencies, as he beats the protagonist Anastasia Steele for his own sexual pleasure (as well as that of Ms. Steele). Despite the politically incorrect plot, display of anti-social personality traits, and male-dominated sexual asymmetry, many women loved the novels which have sold millions of copies around the world. The key ingredient was most likely the fact that Grey’s sadism was mild, controlled, and consensual. He is not a criminal and also, like James Bond, manifests many other qualities such as being handsome, charming, intelligent, and socioeconomically successful (e.g., Jonason et al., 2012; Meston & Buss, 2009; Walter et al., 2020). This example may indicate that many people prefer complex and kaleidoscopic personality types, who possess both some benign and malign traits, despite what they might say in public contexts.

A second aspect to account for is that the undesirability of high levels of Machiavellianism, narcissism, psychopathy, and everyday sadism does not imply that low levels of these traits are necessarily the most optimal balance for individuals and society. Using an analogy of Aristotle (1999a, b), a golden or responsible mean is often preferred over the extremes, both in ethics and politics. Most people prefer “medium” water temperatures when they shower, not the extremely hot or cold ones. Analogously, people often seek the middle ground in politics and ethics. For example, altruistic individuals have to restrain themselves from giving too much of themselves and their resources. Aristotle’s ethics implies that people seek the middle ground (Macintyre, 1998, p. 74). Many people do also score in the middle of the scales in relation to both the Big Five and Dark Tetrad. For example, Davidson (2017) highlights that the ambivert personality type is somewhat of a forgotten concept in personality and social psychology but in fact many people are ambiverts in the sense that their scores are clustered in the middle of Likert scales that cover extraversion/introversion. Perhaps that is even socially and personally beneficial, resembling something of a golden mean and *sophrosyne* (temperance) in the Aristotelian sense.

Moreover, as said, the quantitative evidence from comprehensive personality research also allows for a distinction between grandiose and vulnerable narcissism. It makes more sense to have a medium level of grandiose narcissism and a low level of vulnerable narcissism than the other way around (e.g., Lu et al., 2023; Miller & Maples, 2011). Furthermore, the overlap at the facet and item level allows for more nuanced understandings of the gray areas of the Big Five and Dark Tetrad. Psychopathic personality profiles do partly overlap those of extroverts as they both seek excitement. Yet, no one would deem an extrovert socially undesirable while psychopaths are deemed highly so, in part because of the negative connotations of the Dark Triad concepts (Rauthmann & Kolar, 2012).

Popular culture, and Western culture in general, is brimming with examples of bad boys, bad girls, and adventurous excitement seeking protagonists and supporting characters. One of the latest examples is the Netflix teenage drama *Outer Banks*, in which the young male and female characters constantly do dangerous and reckless things (but with good intentions). Furthermore, bold and “crazy” persons from the real world, like the Australian zoologist and TV personality Steve Irwin (1962–2006), various free climbers and wing suiters are (or, in some cases, were) extremely popular. It seems that the culture in the West does not only cherish specifically narcissistic celebrities but also mildly psychopathic and extrovert behaviors (Boman, 2022; Gentile, 2011; Twenge, 2011; Young & Pinsky, 2006). We may not all want to live fast and die young, but few of us want to be boringly conscientious all year around. Especially as adolescents but perhaps also at later stages in life (Roberts et al., 2008).

Lastly, it is also important to briefly examine the context in which Niccolò Machiavelli (1469–1527) wrote his magnum opus *The Prince* (Machiavelli, 2009) and how the world is constituted today. Machiavelli emphasized that the world is cruel and therefore a leader must appropriate traits such as cunning and aggression and use them both wisely and effectively against his enemies. If the world only consisted of agreeable persons, then this dark direction in leadership theory would not have been necessary for monarchs and political leaders. Aristotle did also understand this a long time before his Renaissance successor. The good citizen, according to Aristotle, is always somewhat out of line with the current regime which tends to be more or less brutal and immoral (Aristotle, 1999b; Pangle, 2013). Machiavelli’s cynical realism has also shaped the political thought of the heterodox Italian Marxist Antionio Gramsci (e.g., Femia, 2005). The current world is, relative to population size, more humane and less war-ridden (Pinker, 2011). Yet, it is a largely market-based world characterized by intense competition between individuals, corporations, and countries (e.g., Baumeister et al., 2017; Gentile, 2011).

Moreover, the current war in Ukraine evokes some of the themes of Machiavelli. Many, at least throughout large swaths of the West, sympathize with Ukraine’s war for independence and territorial integrity which it wages against the Russian federation (Boman, 2023b). Hence, the use of dummy soldiers, vehicles, and weapons – as well as more brutal war tactics – are described in the media as a form of clever cunning rather than malign manipulation (e.g., The Economist, 2023). This is because most people in fact understand that manipulation is crucial and necessary and that the ends justify the means under certain circumstances. Indeed, this is an extreme example which most people will not experience. Nevertheless, it underscores the requirement of “applying” or “appropriating” Machiavellian tactics which in turn demands a latent or learned degree of innate Machiavellianism. For example, in educational contexts such as physical education students may learn how to trick the opposite team or deflect their attention but still abide by the broader rules of the game. In mandatory military service, more serious games are often taught and performed.

### Towards the Gray 9

**Fig. 1:**
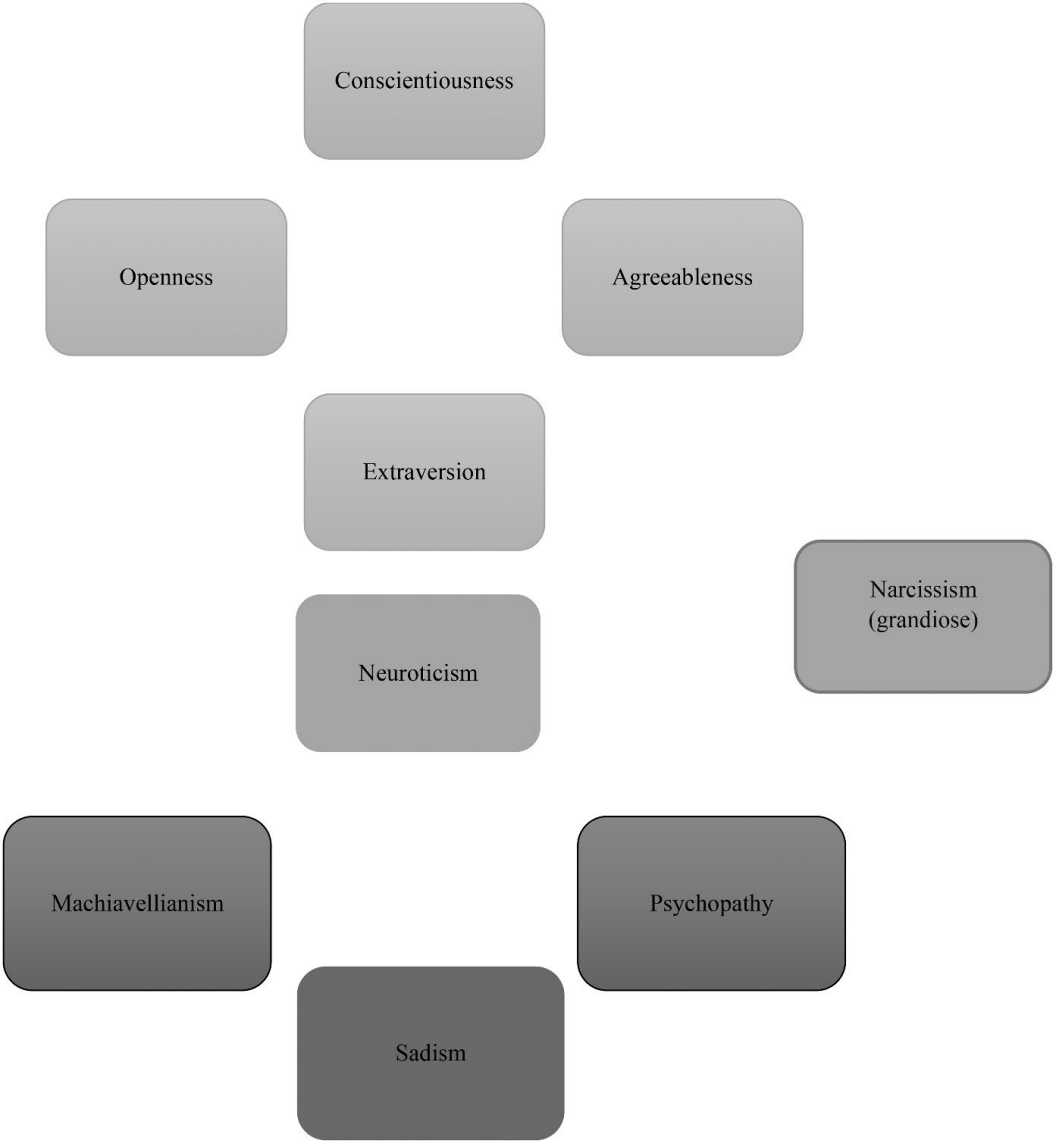
The gray nine

The overall tendency is that high levels of conscientiousness and emotional stability are generally desired. Hence, a person who seeks to have a well-rounded personality profile may aim for a high level of these traits. The rest of the personality traits instead require low or medium levels, alternatively intermediate levels (e.g., low-medium, medium-high, see Table 1 for a summary and Fig. 1 for a graphical illustration). Whilst being a pro-social trait, agreeableness is partly associated with both positive and negative socioeconomic outcomes, as well as negative subjective measures of wellbeing (e.g., Duckworth et al., 2012). This might, in part, be because less agreeable persons take advantage of their more agreeable counterparts. It might also be because many of the pro-social job types, often funded by the public sector and not particularly lucrative, are overrepresented by agreeable individuals (typically females) (e.g., Alderotti et al., 2023). In a world in which many people are not agreeable, it is necessary to learn to be both agreeable and disagreeable depending on the circumstances. This is one of the old lessons of Machiavelli’s work and it is still relevant to a degree.

**Table 1:** Socially and personally desired levels

Trait	Socially and personally desired level
Openness to experience	Medium
Conscientiousness	High (not extreme)
Extraversion	Medium
Agreeableness	Medium
Neuroticism	Low
Narcissism (grandiose)	Medium
Machiavellianism	Low-medium
Psychopathy	Low-medium
Sadism	Low

A Dark Tetrad trait such as grandiose (i.e., overt) narcissism seems to be relatively neutral in the eyes of others (Rauthmann & Kolar, 2012) and has many personal benefits such as an inverted relationship with depressive symptoms. Narcissists of the vulnerable variety do not manifest any such positive socioeconomic or subjective associations (Miller & Maples, 2011; Papageorgiou et al., 2019). Hence, the Gray 9, as I succinctly label the combination of the Big Five and Dark Tetrad traits, does not include vulnerable narcissism. Instead, while relevant for clinical literature, this does rather belong to the neuroticism trait. If one insists on the inclusion of measures of vulnerable narcissism as a narcissism construct, then these could be considered A and B types of narcissism within the Gray 9. Moreover, grandiose narcissism, due to its generally weak correlations with, in particular, everyday sadism, might be omitted from the Dark Tetrad. Instead, novel research shows that Machiavellianism, psychopathy, and everyday sadism constitute a more uniform constellation of dark gray traits (e.g., Bonfa-Araujo et al., 2022). As such they would, preferably, make up a new Dark Triad. Grandiose narcissism belongs in the middle of the light gray Big Five traits and the revised dark gray Dark Triad.

Regarding the Dark Tetrad it is pertinent to accentuate that it is specifically the low to medium scores that cancel out the dark and socially undesirable associations. Low or medium scores in grandiose narcissism cancel out (complete) disagreeableness whereas higher scores do not. That is also the case with Machiavellianism and psychopathy, although relatively lower scores on these darker traits appear to be constituted as the more socially and personally optimal levels. As everyday sadism is, except for perhaps some contextual sexual benefits (e.g., see *50 Shades of Grey*), not linked to any personally and/or socially beneficial outcomes this trait may optimally be as low as possible.

### Parallel Personality Patterns: Good/Bad Instead of High, Low, and Moderate

Parallelization theory (Boman, 2021, 2024) implies that societies and the people who inhabit them are often too complex for a single predominant pattern to emerge. Instead, many processes, in a paradoxical fashion, occur simultaneously as interacting, opposite forces. For example, cognitive ability at the individual level is influenced by both biological (e.g., genes) and environmental factors (e.g., education, effort, family structure). An average cognitive ability score can increase even as people who on average have higher cognitive ability (e.g., people with higher education) have fewer children in some contexts. Cognitive ability levels at a broader social level can also decrease in some contexts, despite people with higher cognitive ability having more children, on average. That indicates parallel and complex biological and social processes that appear contradictory at first glance but whose underlying structures may be disentangled with proper interpretations and meaningful data (Boman, 2024).

Moreover, people can, in certain situations, even be both happy and unhappy simultaneously such as when they graduate from high school (Larsen et al., 2001). Countries such as Georgia and Ukraine are becoming Westernized and Russified in parallel, which seems contradictory at first glance (Boman, 2024).

Regarding personality clusters such as the FFM and Dark Tetrad, it seems proper to regard many of the traits which make up these two constructs as not just relatively good or bad on a continuum, and thus with relative levels of appropriate degrees (e.g., high conscientiousness, moderate agreeableness), but also simultaneously good/bad. Not everything in life is about finding the proper balance but about accepting the associated trade-offs (Seligman, 2002). The same goes for personality traits (e.g., Carter et al., 2018; Papageorgiou et al., 2019). An agreeable person will be judged favorably by society in many regards, whereas Machiavellians and psychopaths will not (Rauthmann & Kolar, 2012). However, an agreeable person might also be taken advantage of and have a relatively harder time excelling in competitive work environments (e.g., Duckworth et al., 2012; Lu et al., 2023). Hence, it is simultaneously good and bad. Furthermore, an extremely conscientious person will likely have important advantages in many contexts (e.g., education, earnings) but also potentially suffer from a severe workload (Boman, 2020; Coleman et al., 2022). Grandiose narcissism is associated with higher earnings and lower stress (Lu et al., 2023; Papageorgiou et al., 2019) but also social and personal disadvantages such as relationship problems (Muris et al., 2017). Hence, it appears good/bad in parallel.

### Concluding Remarks

While it is fairly obvious that conscientiousness and emotional stability (i.e., low neuroticism) are socially and individually desirable outcomes, as they are associated with, for instance, higher academic achievement and earnings as well as a stable social life (Barrick & Mount, 2012; Mammadov, 2022), a number of caveats are important to consider. It is, for instance, wise to recall the old personality/situation debate (e.g., Almlund et al., 2011; Goldberg, 1990). In certain situations and contexts, all people experience stress, neurotic tendencies, and non-conscientious behavior. These are natural responses and coping mechanisms related to life trauma (e.g., the death of beloved ones, relationship breakups, social stigma, or uprisings) and changing circumstances. Large shares of females experience for example moodiness and irritability prior and during menstruation. Should females be blamed for internal biological hormonal processes? Should ordinary people be blamed for not being productive and stable 365 days of each year? Should introverts be blamed for not being adequately assertive and gregarious? Particularly as personality traits, like intelligence, are partly heritable (John & Srivastava, 2009).

Moreover, and as stated above, there might be a trade-off between very high conscientiousness and moderate excitement seeking (a facet of extraversion), of which the latter is probably required to experience interesting events in life. As personality traits are partially changing throughout the life course, it is probably the case that excitement seeking is higher in adolescents and younger adults and then gradually decreases later in life whereas agreeableness, conscientiousness and emotional stability typically increase (Caspi et al., 2005). If younger men and women, for example, do not travel to other countries, to have fun and to cultivate themselves, then life becomes restricted. It is also difficult to appropriate a “global citizenship” (e.g., Boman & Mosesson, 2023) if one does not take calculated risks and expands one’s worldview beyond the everyday habits of the workplace or one’s regular place of sojourn. Such pursuits require both some degree of extraversion and openness to experience.

While we cannot prove that general correlations imply causal links at the individual level, earlier research results and theoretical foundations inform us of the relations and dynamics between personality profiles and behavioral repertoires. These could help us to understand a world and the people who inhabit it, which are not unitarily bright or dark but often characterized by various shades of gray, as well as parallelization of good/bad with having, for example, high levels of agreeableness and narcissism. Hence, a well-rounded personality profile should be balanced and varied and not necessarily shy from the darker sides of reality. To the very least, the broader society and education should emphasize the complexity of personality theories such as FFM and Dark Tetrad. Perhaps this should also be underlined at appropriate age levels in national curricular documents around the world.

The current synthesis has many limitations. The literature review is not exhaustive but as a theoretical synthesis it may be regarded as at least sufficiently extensive. Moreover, there are, indeed, other pertinent personality traits and constructs that are derived from the clinical literature (e.g., Rosenfeld, 1990) and personality and social psychology than those that constitute the Big Five and the Dark Tetrad. For example, grit is often seen as an important contribution to personality psychology and other strands of psychology (e.g., Duckworth & Quinn, 2009) but it adds very limited incremental validity beyond conscientiousness (Muenks et al., 2017; Ponnock et al., 2020). Many may not agree with the proposed separation of grandiose narcissism from other “dark” traits, as well as from vulnerable narcissism. In that case, a researcher may use, for instance, both the BFI-10, SD4, and the Personality Inventory for DSM-5 (Miller et al., 2013).

Be that as it may, I hope that the current examination could stimulate further scholarly debate and a more nuanced understanding of our societies, human behaviors, and real-life personality profiles, as well as those that are linked to the realm of popular culture. While most standard handbooks on personality psychology stress the upsides and downsides of different personality traits, there is yet no conceptual framework that regards these as different shades of gray or simultaneously good/bad. The gray nine and parallelization of personality patterns constitute an alternative conceptual outlook on these broader theories in psychology.

## Data Availability

No datasets were generated or analysed during the current study.
