# Correction: The Gray Nine and Parallel Personality Patterns: Big Five, Dark Tetrad, and a “Well-Rounded Personality”

**DOI:** 10.1007/s12124-024-09855-7

**Published:** 2024-07-02

**Authors:** Björn Boman

**Affiliations:** Stockholm University, 114 19, Stockholm, Sweden

**Correction: Integrative Psychological and Behavioral Science**

10.1007/s12124-024-09842-y

The original version of this article unfortunately contained a mistake.

In the fourth paragraph under the header Personality, Popular Culture, and Politics, the sentence:

Furthermore, the overlap at the facet and item level overlaps allows for more nuanced understandings of the gray areas of the Big Five and Dark Tetrad. Psychopathic personality profiles do partly overlap those of extroverts as they both seek excitement.

Should read:

Furthermore, the overlap at the facet and item level allows for more nuanced understandings of the gray areas of the Big Five and Dark Tetrad. Psychopathic personality profiles do partly overlap those of extroverts as they both seek excitement.

The original article has been updated.

